# Export of polybasic motif–containing secretory proteins BMP8A and SFRP1 from the endoplasmic reticulum is regulated by surfeit locus protein 4

**DOI:** 10.1016/j.jbc.2022.102687

**Published:** 2022-11-09

**Authors:** Xiao Tang, Tingxuan Wang, Yusong Guo

**Affiliations:** 1 College of Life Sciences, Anhui Normal University, Wuhu, Anhui, China; 2 Division of Life Science, The Hong Kong University of Science and Technology, Hong Kong, China; 3 Hong Kong University of Science and Technology Shenzhen Research Institute, Shenzhen, China; 4 Southern Marine Science and Engineering Guangdong Laboratory (Guangzhou), Guangzhou, China

**Keywords:** COPII, SURF4, cargo sorting, secretion, ER export, ATPrS, ATP regeneration system, BMP8A, bone morphogenetic protein 8A, COPII, coat protein complex II, CW, Cardin–Weintraub, Dhh, desert hedgehog, DhhN, N-terminal fragment of Dhh, EGFP, enhanced GFP, ER, endoplasmic reticulum, ERES, ER exit sites, ERGIC, ER-Golgi intermediate compartment, Erv, ER vesicle, GST, glutathione S-transferase, Ihh, Indian hedgehog, IhhN, N-terminal fragment of Ihh, RLC, rat liver cytosol, RUSH, Retention Using Selective Hook, SBP, streptavidin-binding peptide, SFRP1, secreted frizzled-related protein 1, Shh, sonic hedgehog, SURF4, surfeit locus protein 4

## Abstract

In the conventional secretory pathway, cargo receptors play important roles in exporting newly synthesized secretory proteins from the endoplasmic reticulum (ER). We previously showed that a cargo receptor, surfeit locus protein 4 (SURF4), promotes ER export of a soluble signaling molecule, sonic hedgehog, *via* recognizing the polybasic residues within its Cardin–Weintraub motif. In addition to sonic hedgehog, we found 30 more secretory proteins containing the polybasic motif (K/R)(K/R)(K/R)XX(K/R)(K/R), but whether SURF4 plays a general role in mediating ER export of these secretory proteins is unclear. Here, we analyzed the trafficking of four of these secretory proteins: desert hedgehog, Indian hedgehog, bone morphogenetic protein 8A (BMP8A), and secreted frizzled-related protein 1 (SFRP1). We found that the polybasic motifs contained in these cargo proteins are important for their ER export. Further analyses indicated that the polybasic motifs of BMP8A and SFRP1 interact with the triacidic motif on the predicted first luminal domain of SURF4. These interactions with SURF4 are essential and sufficient for the ER-to-Golgi trafficking of BMP8A and SFRP1. Moreover, we demonstrated that SURF4 localizes at a subpopulation of ER exit sites to regulate the ER export of its clients. Taken together, these results suggest that SURF4 is recruited to specific ER exit sites and plays a general role in capturing polybasic motif–containing secretory cargo proteins through electrostatic interactions.

Newly synthesized secretory proteins need to be released into the extracellular space through the biosynthetic pathway to perform their biological functions. The endoplasmic reticulum (ER) is the first station that cargo proteins encounter. At the ER, many cargo proteins bearing specific sorting motifs are recognized by coat protein complex II (COPII) to be enriched in transport vesicles. This process is important to secure high efficiency and accuracy of cargo trafficking (1, 2). Transmembrane cargo proteins can be directly recognized by the COPII subunit, whereas soluble cargo proteins in the ER lumen can be indirectly linked to the cytosolic COPII coat through interaction with transmembrane cargo receptors.

Although fundamentally important, only a handful of cargo receptors at the ER have been characterized, including ER-Golgi intermediate compartment 53 kDa protein (ERGIC-53), p24 family proteins, and some of the abundant ER vesicle (Erv) proteins (2). They all contain COPII-interacting motifs and COPI-interacting motifs in their cytosolic domain and recognize cargo proteins through various sorting signals (2). As such, these cargo receptors facilitate COPII-mediated packaging of cargo proteins at the ER and are retrieved from the Golgi to the ER through the COPI-dependent pathway. ERGIC-53 recognizes N-linked glycoproteins in the ER lumen, including coagulation factor V/VIII, cathepsin C/Z, and α1-antitrypsin (3, 4). The p24 proteins assemble into heterooligomers to regulate the ER export of glycosylphosphatidylinositol-anchored proteins, such as gas1p and secretory invertase (2). Many yeast Erv proteins are shown to function as cargo receptors at the ER. Erv14p regulates the ER export of Gurken and Axl2p (5, 6). Erv26p is important for the ER exit of Ktr3p mannosyltransferase and alkaline phosphatase (7). Erv29p is required for the packaging of guanine nucleotide-binding protein alpha-6 subunit (gpαf), carboxypeptidase Y, and proteinase A into COPII vesicles (2).

Surfeit locus protein 4 (SURF4) is the ortholog of Erv29p in mammals. It regulates the ER export of secreted proteins proprotein convertase subtilisin/kexin type 9 (PCSK9), lipoprotein, C-X-C motif chemokine 9 (CXCL9), and α1-anti-trypsin (α1AT) and lysosomal proteins prosaposin and progranulin (8, 9, 10, 11, 12). SURF4 participates in ER exit site (ERES) organization and tubular ERGIC formation (10, 13). It also plays a role in the retrograde trafficking of stimulator of interferon genes at the Golgi apparatus (14). SURF4 has been reported to recognize the amino-terminal tripeptide motifs of soluble cargo proteins with high affinity to the hydrophobic-proline-hydrophobic (φ-P-φ) motif (15). We recently reported that SURF4 regulates the ER exit of sonic hedgehog (Shh) by interacting with the polybasic residues within the Cardin–Weintraub (CW) motif on Shh (16). Given this context, we hypothesized that SURF4 would play a general recognition mechanism for the ER-to-Golgi transport of other secretory proteins bearing similar polybasic motifs.

Here, using the Retention Using Selective Hook (RUSH) assay (17), we found that the polybasic motif, (K/R)(K/R)(K/R)XX(K/R)(K/R), in four secretory proteins, including desert hedgehog (Dhh), Indian hedgehog (Ihh), bone morphogenetic protein 8A (BMP8A), and secreted frizzled-related protein 1 (SFRP1), are essential for their ER export. Furthermore, we showed that SURF4 is located at a subpopulation of ERES and recognizes two of these proteins, BMP8A and SFRP1, through electrostatic interactions between a triacidic motif in SURF4 and the polybasic cargo sorting motif. These analyses expand our understandings of the functional roles of SURF4 in mediating exporting secretory proteins out of the ER.

## Results

### ER-to-Golgi transport of Dhh, Ihh, BMP8A, and SFRP1 depends on their polybasic motifs

**Figure 1:**
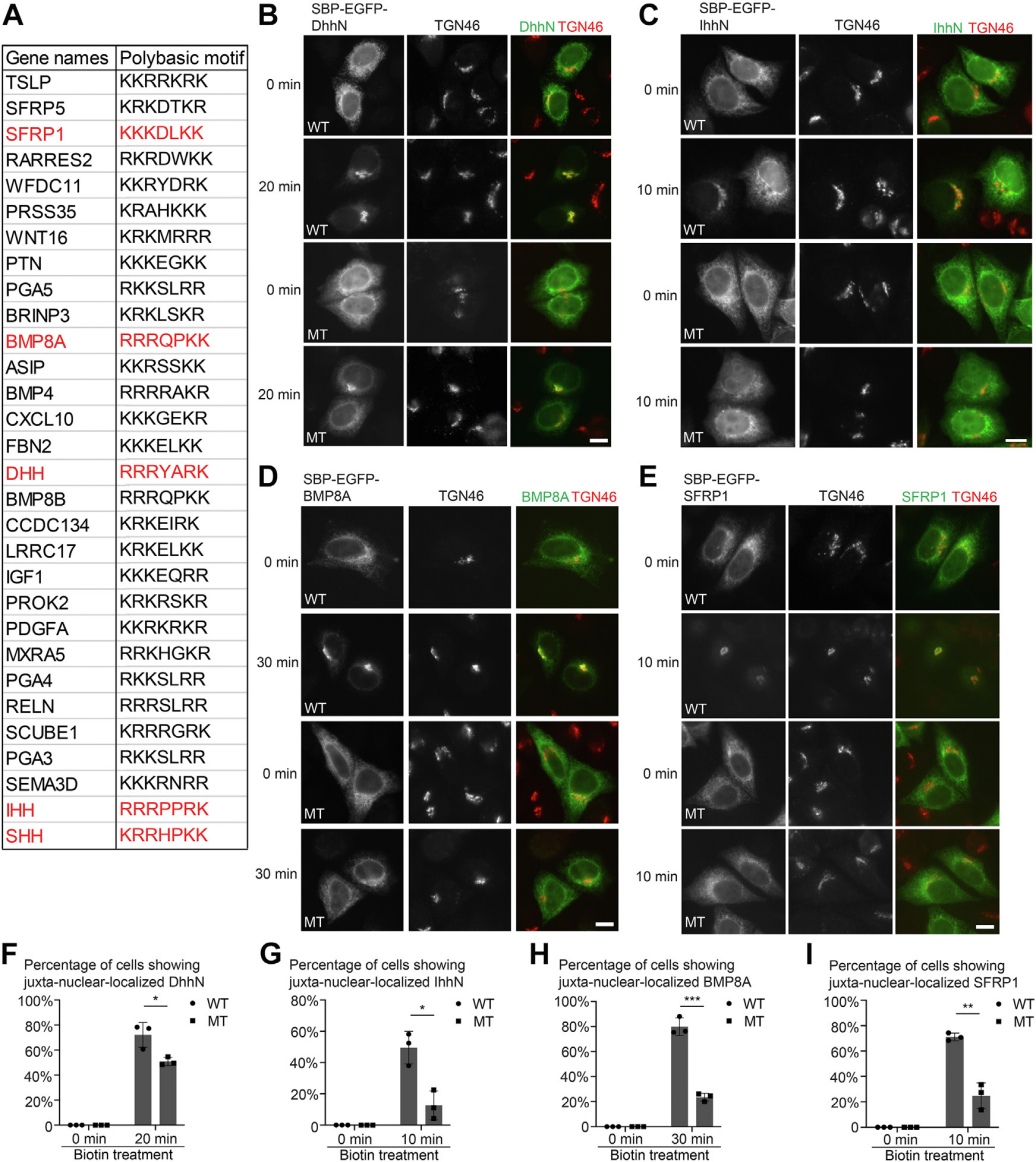
**The polybasic motif is important for the ER-to-Golgi transport of DhhN, IhhN, BMP8A, and SFRP1.***A*, list of identified secretory proteins containing the polybasic motif. *B–E*, HeLa cells were transfected with plasmids encoding Str-KDEL and WT or mutant versions of SBP-EGFP-DhhN (*B*), SBP-EGFP-IhhN (*C*), SBP-EGFP-BMP8A (*D*), and SBP-EGFP-SFRP1 (*E*). Day 1 after transfection, the localization of the different versions of RUSH constructs was analyzed after incubation with biotin for the indicated time (The scale bar represents 10 μm). Magnification, 63×. *F–I*, quantifications of the percentage of cells showing juxta-nuclear–accumulated EGFP signal after incubation with biotin for the indicated time (mean ± SD; n = 3; >50 cells counted for each time point). ∗*p* < 0.05; ∗∗*p* < 0.01; ∗∗∗*p* < 0.001. BMP8A, bone morphogenetic protein 8A; Dhh, desert hedgehog; DhhN, N-terminal fragment of Dhh; ER, endoplasmic reticulum; EGFP, enhanced GFP; Ihh, Indian hedgehog; IhhN, N-terminal fragment of Ihh; RUSH, Retention Using Selective Hook; SBP, streptavidin-binding peptide; SFRP1, secreted frizzled-related protein 1.

We previously reported that cargo receptor SURF4 regulates ER export of Shh by interacting with the basic residues within its CW motif (16). The CW motif of Shh contains basic residues, and these residues showed a consensus sequence of (K/R)(K/R)(K/R)XX(K/R)(K/R). Here, we further retrieved annotated secretory proteins containing this consensus sequence based on GenomeNet database and obtained a list of 30 secretory proteins (18, 19) (Fig. 1*A*). To test whether SURF4 plays a general role in regulating ER export of these polybasic motif–containing secretory proteins, we analyzed the ER export of four secretory proteins in the list (Dhh, Ihh, BMP8A, and SFRP1). Dhh and Ihh are members of the Hh family, and they function in testis organogenesis and chondrocytes, respectively (20, 21). BMP8A plays a role in calcium regulation and bone homeostasis (22). SFRP1 is a secreted antagonist of Wnt signaling (23).

We utilized a RUSH transport assay to analyze the trafficking of these cargo proteins in a synchronized manner (17). HeLa cells were transfected with plasmids encoding BMP8A, SFRP1, the N-terminal fragment of Dhh or Ihh (DhhN or IhhN) fused downstream of enhanced GFP (EGFP), and the streptavidin-binding peptide (SBP) that had an N-terminal signal peptide derived from IL-2 (SBP-EGFP-DhhN, SBP-EGFP-IhhN, SBP-EGFP-BMP8A, or SBP-EGFP-SFRP1). The plasmids also encode streptavidin fused to a C-terminal ER retention signal (Lys-Asp-Glu-Leu; Str-KDEL). In HeLa cells transfected with these plasmids, SBP-fused proteins are retained in the ER as a result of the binding between streptavidin and SBP. When cells are incubated with biotin, SBP is released from streptavidin, thereby releasing the RUSH cargo proteins from the ER. Around 10 to 30 min after biotin treatment, the RUSH cargo proteins were localized at the juxta-nuclear area in the majority of cells, colocalized with a Golgi marker TGN46 (Fig. 1, *B–E* and quantifications in Fig. 1, *F–I*). These results indicate that the RUSH assay is sufficiently robust to analyze the kinetics of ER-to-Golgi transport of cargo proteins DhhN, IhhN, BMP8A, and SFRP1.

We then substituted the charged amino acids in the polybasic motifs of these cargo proteins to alanines. We found that the percentages of cells showing juxta-nuclear–localized mutated forms of DhhN, IhhN, and BMP8A were significantly reduced compared with WT cargo proteins (Fig. 1, *B*–*D* and quantifications in Fig. 1, *F*–*H*). Unexpectedly, the percentage of cells showing juxta-nuclear–localized mutated pattern of the mutant form of SFRP1 was similar to WT SFRP1 (Fig. S1, *B–M* and quantification in Fig. S1*P*). We noticed a series of additional lysine residues in the vicinity of the polybasic motif on SFRP1 (Fig. S1*A*). These additional lysine residues and the (K/R)(K/R)(K/R)XX(K/R)(K/R) motif in SFRP1 are conserved across species (Fig. S1*A*) and may play redundant roles in mediating ER export of SFRP1. To test this possibility, we generated another mutated form of SFRP1 in which all these positively charged residues of SFRP1 were substituted to alanine (SBP-EGFP-SFRP1^MT^). We found that SBP-EGFP-SFRP1^MT^ showed significant kinetic delay of ER-to-Golgi transport (Fig. 1*E* and quantification in Fig. 1*I*). These results suggest that the polybasic motif is important for the ER-to-Golgi transport of DhhN, IhhN, BMP8A, and SFRP1.

### SURF4 regulates the ER-to-Golgi trafficking of Dhh, Ihh, BMP8A, and SFRP1

**Figure 2:**
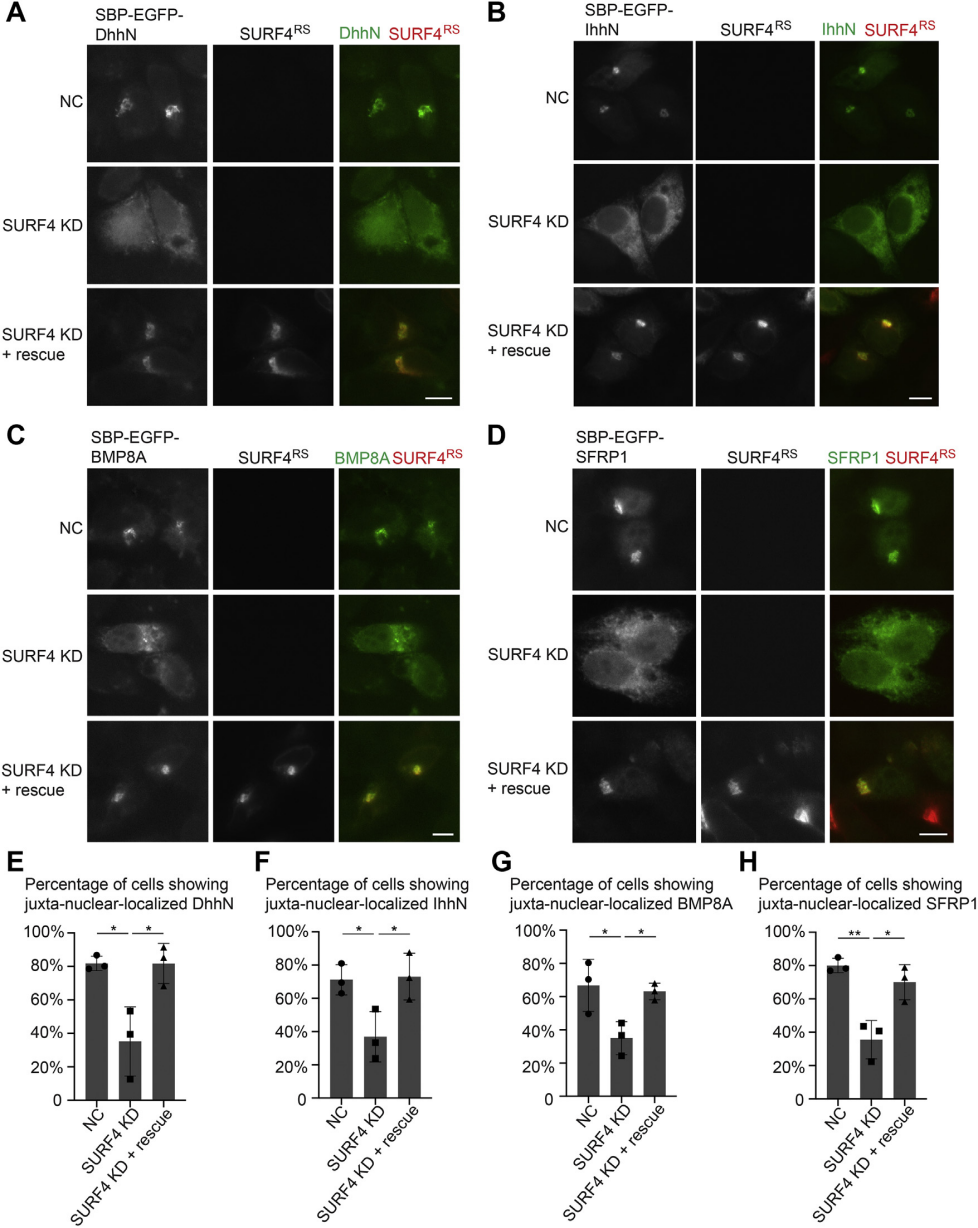
**SURF4 regulates ER-to-Golgi trafficking of DhhN, IhhN, BMP8A, and SFRP1.***A–D*, HeLa cells were transfected with negative control (NC) siRNA or siRNA against SURF4. At 48 h after transfection, cells were retransfected with plasmids encoding Str-KDEL_SBP-EGFP-DhhN (*A*), Str-KDEL_SBP-EGFP-IhhN (*B*), Str-KDEL_SBP-EGFP-BMP8A (*C*), and Str-KDEL_SBP-EGFP-SFRP1 (*D*) or cotransfected with siRNA-resistant SURF4^RS^-HA and RUSH construct of DhhN (*A*), IhhN (B), BMP8A (*C*), and SFRP1 (*D*). On day 3 after knockdown, cells were incubated with biotin for 15 min (*A–B*), 30 min (*C*), or 8 min (*D*), and the localization of DhhN, IhhN, BMP8A, and SFRP1 was analyzed (The scale bar represents 10 μm). Magnification, 63×. *E*–*H*, quantifications of the percentage of cells showing juxta-nuclear-accumulated EGFP signal (mean ± SD; n = 3; >50 cells counted for each group). ∗*p* < 0.05; ∗∗*p* < 0.01. BMP8A, bone morphogenetic protein 8A; Dhh, desert hedgehog; DhhN, N-terminal fragment of Dhh; ER, endoplasmic reticulum; EGFP, enhanced GFP; Ihh, Indian hedgehog; IhhN, N-terminal fragment of Ihh; RUSH, Retention Using Selective Hook; SBP, streptavidin-binding peptide; SFRP1, secreted frizzled-related protein 1; SURF4, surfeit locus protein 4.

SURF4 regulates the ER-to-Golgi trafficking of Shh (16). To test whether SURF4 is also crucial for the ER export of other polybasic motif–containing secretory cargo clients, we performed an siRNA knockdown experiment to reduce the expression of SURF4 (16). We analyzed the impact on ER export of IhhN, DhhN, BMP8A, and SFRP1. We found that knockdown of SURF4 significantly reduced the efficiency of ER-to-Golgi transport of the RUSH construct of these cargo proteins after biotin treatment (Fig. 2, *A–D* and quantifications in Fig. 2, *E–H*). These defects were rescued by the expression of an siRNA-resistant construct of SURF4 (SURF4^RS^-HA; Fig. 2, *A*–*D* and quantifications in Fig. 2, *E*–*H*). These analyses indicate that SURF4 regulates the ER-to-Golgi trafficking of other polybasic motif–containing cargo proteins, including IhhN, DhhN, BMP8A, and SFRP1.

### The triacidic motif in SURF4 interacts with the polybasic motifs of BMP8A and SFRP1

**Figure 3:**
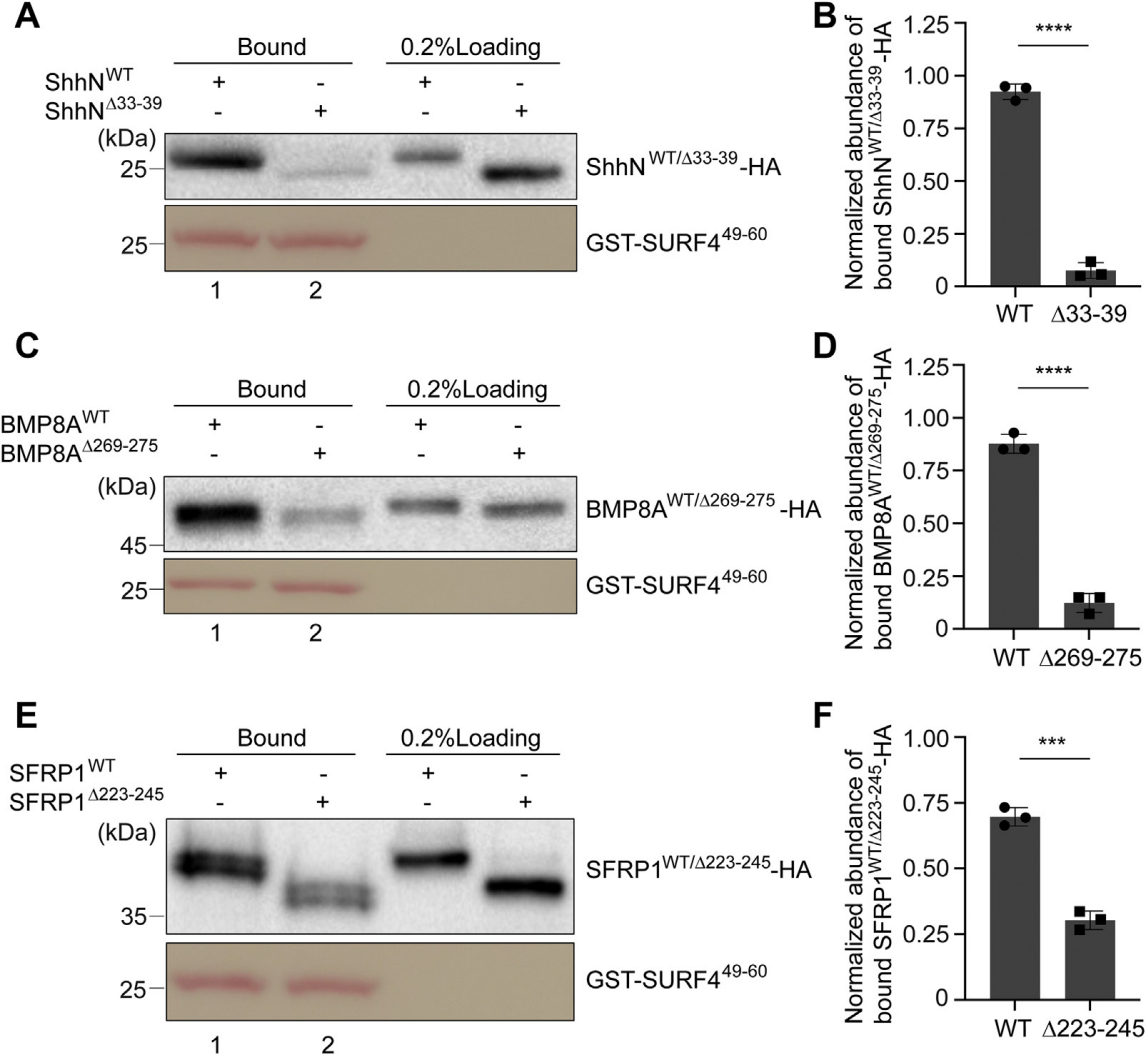
**The predicted first luminal loop of SURF4 interacts with the polybasic motif of SFRP1 and BMP8A.***A*, *C* and *E*, purified GST-tagged human SURF4^49–60^ was incubated with lysates from HEK293T cells transfected with plasmids encoding WT or polybasic motif–depleted ShhN-HA (*A*), BMP8A-HA (*C*), and SFRP1-HA (*E*). After incubation, the bound proteins were analyzed by immunoblotting with anti-HA antibodies. *B*, *D* and *F*, normalized abundances of ShhN-HA (*B*), BMP8A-HA (*D*), or SFRP1-HA (*F*) bound to GST-SURF4^49–60^ were quantified (mean ± SD; n = 3). The abundance of bound proteins was normalized to the bait protein GST-SURF4^49–60^, and this value was then normalized to the sum of the normalized abundance of WT and mutant versions of cargo proteins bound to GST-SURF4^49–60^ in each experimental group. ∗∗∗*p* < 0.001; ∗∗∗∗*p* < 0.0001. BMP8A, bone morphogenetic protein 8A; SFRP1, secreted frizzled-related protein 1; Shh, sonic hedgehog; SURF4, surfeit locus protein 4.

Given that Dhh and Ihh are Hh family members, bearing high homology with Shh, we focused on BMP8A and SFRP1 in the following analyses. The polybasic motif of Shh directly interacts with the AlphaFold-predicted first luminal loop of SURF4 (SURF4^49–60^) (16) (Fig. 8*N’*). To test whether this fragment of SURF4 interacts with BMP8A and SFRP1, we performed glutathione S-transferase (GST) pull-down experiments using recombinant purified GST-tagged SURF4^49–60^ (GST-SURF4^49–60^) and lysates from HEK293T cells expressing 3 × HA-tagged WT ShhN, BMP8A, or SFRP1 (ShhN^WT^-HA, BMP8A^WT^-HA, or SFRP1^WT^-HA). We found that GST-SURF4^49–60^ interacted with these HA-tagged proteins in cell lysates (Fig. 3, *A*, *C* and *E*, lane 1). Deleting the polybasic motifs in ShhN and BMP8A significantly reduced the abundance of ShhN and BMP8A bound to GST-SURF4^49–60^ (Fig. 3, *A* and *C*, compare lanes 1 and 2, and quantifications in Fig. 3, *B* and *D*). Deleting the (K/R)(K/R)(K/R)XX(K/R)(K/R) motif in SFRP1 did not affect the interaction between SFRP1 and GST-SURF4^49–60^ (Fig. S1*N*, compare lanes one and two and quantification in Fig. S1*O*). In contrast, deleting the sequence of SFRP1 containing the (K/R)(K/R)(K/R)XX(K/R)(K/R) motif and lysine residues in the vicinity of this motif (aa: 223–245) significantly reduced the abundance of bound SFRP1 (Fig. 3*E*, compare lanes 1 and 2, and quantification in Fig. 3*F*). These analyses were consistent with our immunofluorescence analyses, suggesting that two stretches of the polybasic motif play redundant roles to allow SURF4 to capture SFRP1. These results suggest that SURF4 captures BMP8A and SFRP1 through their polybasic motifs.

**Figure 4:**
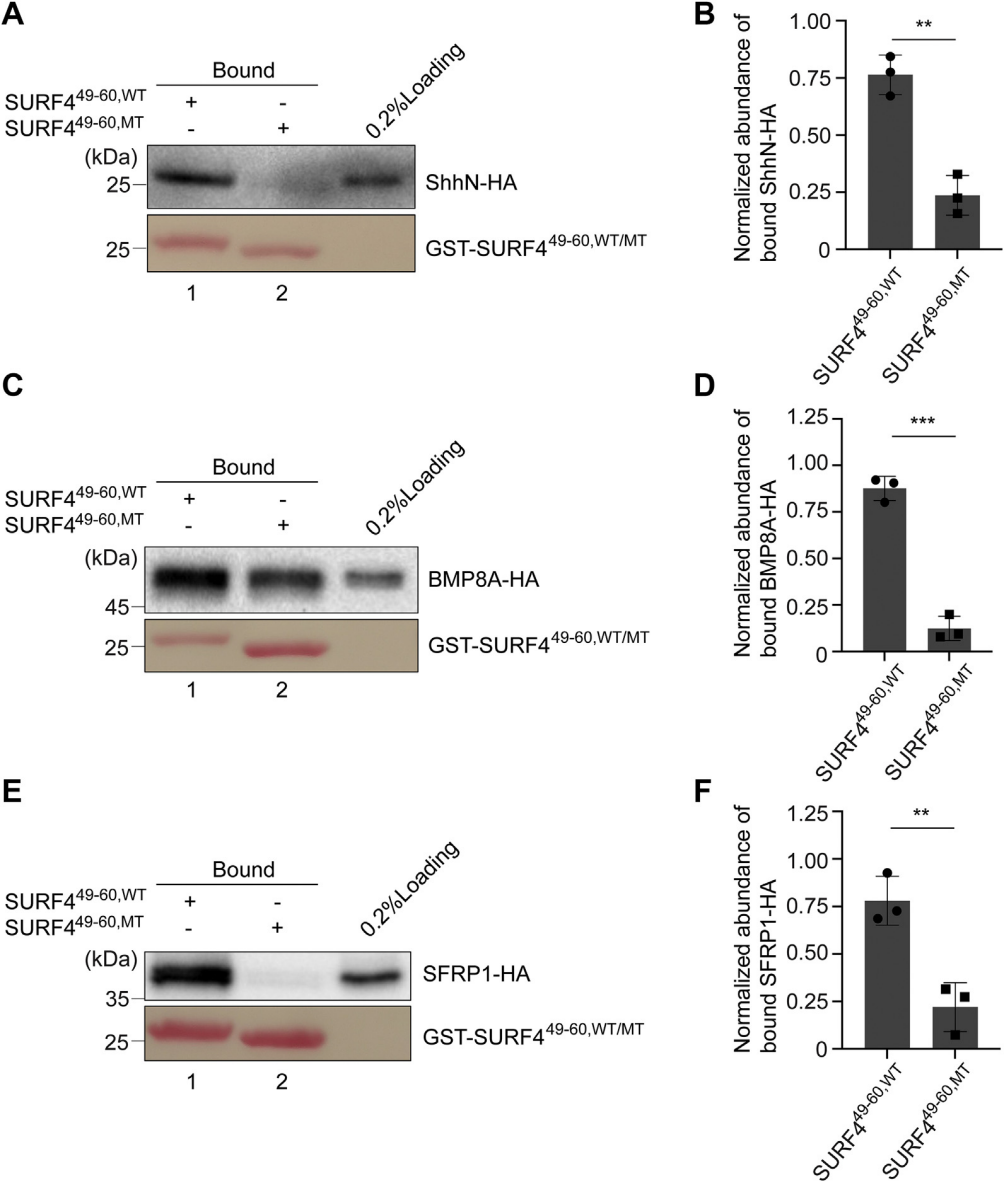
**SURF4 recognizes BMP8A and SFRP1 through the triacidic motif in its predicted first luminal loop.***A*, *C* and *E*, purified GST-tagged human WT or triacidic motif–mutated SURF4^49–60^ was incubated with lysates from HEK293T cells transfected with plasmids encoding ShhN-HA (*A*), BMP8A-HA (*C*), or SFRP1-HA (*E*). After incubation, the bound proteins were analyzed by immunoblotting with anti-HA antibodies. *B*, *D* and *F*, normalized abundances of ShhN-HA (*B*), BMP8A-HA (*D*), or SFRP1-HA (*F*) bound to GST-SURF4^49–60,WT^ or GST-SURF4^49–60,MT^ were quantified (mean ± SD; n = 3). The abundance of bound proteins was normalized to the corresponding bait protein, and this value was then normalized to the sum of the normalized abundance of cargo proteins bound to GST-SURF4^49–60,WT^ and GST-SURF4^49–60, MT^ in each experimental group. ∗∗*p* < 0.01; ∗∗∗*p* < 0.001. BMP8A, bone morphogenetic protein 8A; SFRP1, secreted frizzled-related protein 1; Shh, sonic hedgehog; SURF4, surfeit locus protein 4.

The first luminal loop of SURF4 (aa: 49–60) was predicted by AlphaFold to form a helix with three negatively charged residues that point toward the lumen (residues E50, D53, and D56) (Fig. 8*N’*) (16). To test whether these residues are critical for the interaction with the polybasic motifs on BMP8A and SFRP1, we substituted E50, D53, and D56 of GST-SURF4^49–60^ to alanine (GST-SURF4^49–60,MT^). We found that the abundance of ShhN-HA, BMP8A-HA, and SFRP1-HA that bound to SURF4^49–60,WT^ was significantly higher than that bound to SURF4^49–60,MT^ (Fig. 4, *A*, *C* and *E*, compare lanes 1 and 2, and quantifications in Fig. 4, *B*, *D* and *F*). These analyses suggest that the polybasic motifs of BMP8A and SFRP1 are recognized by the triacidic motif on the predicted first luminal loop of SURF4 through electrostatic interactions.

### ER export of BMP8A and SFRP1 depends on the triacidic motif in the predicted first luminal loop of SURF4

**Figure 5:**
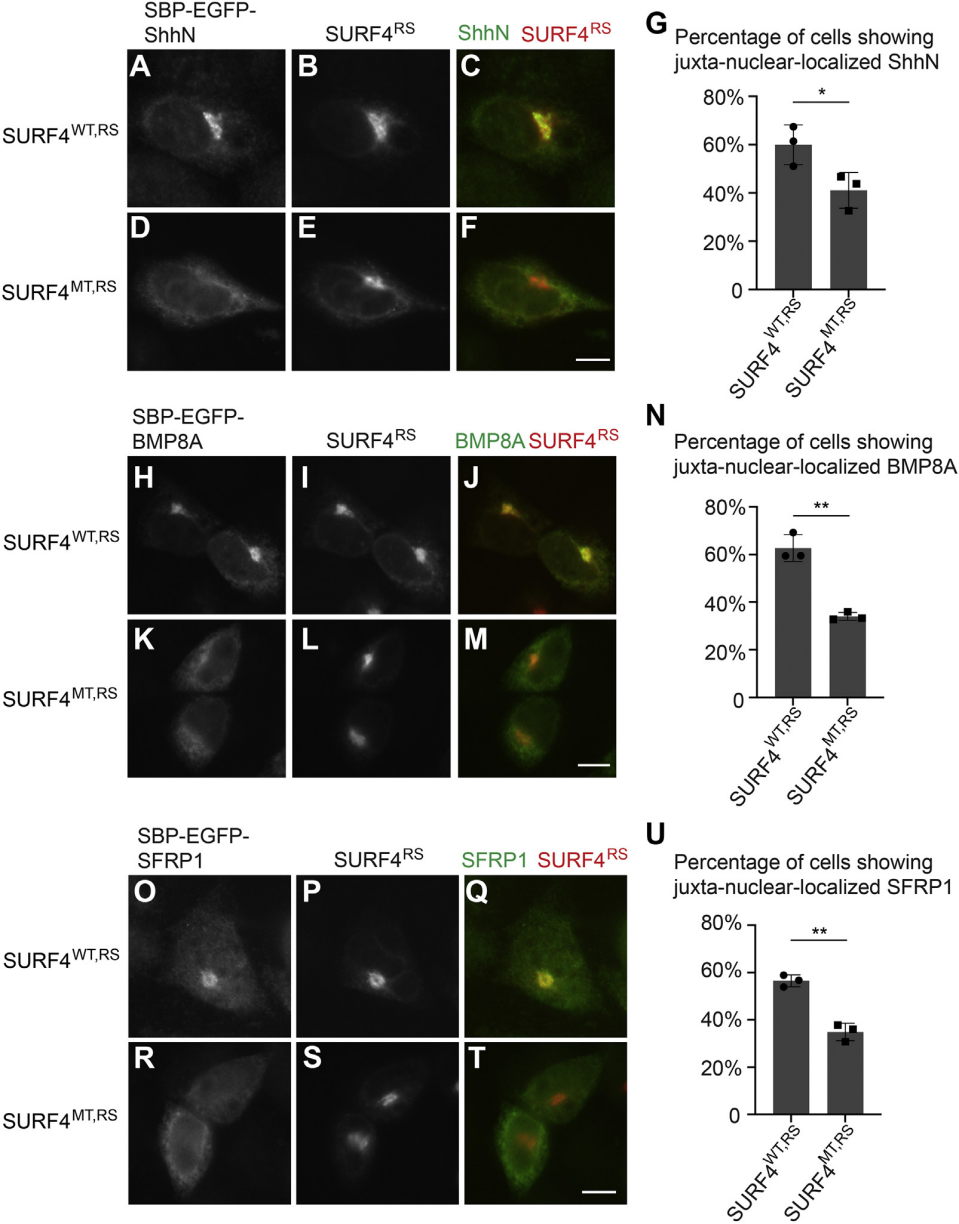
**The triacidic motif on SURF4 is important for the ER-to-Golgi transport of ShhN, BMP8A, and SFRP1.***A*–*F*, *H*–*M* and *O*–*T*, HeLa cells were transfected with siRNA against SURF4. At 48 h after transfection, cells were cotransfected with plasmids encoding the indicated RUSH constructs and siRNA-resistant SURF4^WT,RS^-HA (*A*–*C*, *H*–*J* and *O*–*Q*) or siRNA-resistant, triacidic motif–mutated SURF4^MT,RS^-HA (*D*–*F*, *K*–*M*, and *R*–*T*). On day 3 after knockdown, cells were incubated with biotin for 15 min (*A*–*F*), 30 min (*H*–*M*), or 8 min (*O*–*T*), and the localization of ShhN, BMP8A, and SFRP1 was analyzed (The scale bar represents 10 μm). Magnification, 63×. *G*, *N*, and *U*, quantifications of the percentage of cells showing juxta-nuclear–accumulated EGFP signal (mean ± SD; n = 3; >50 cells counted for each group). ∗*p* < 0.05; ∗∗*p* < 0.01. BMP8A, bone morphogenetic protein 8A; EGFP, enhanced GFP; ER, endoplasmic reticulum; RUSH, Retention Using Selective Hook; Shh, sonic hedgehog; SURF4, surfeit locus protein 4.

We then tested whether the E50, D53, and D56 residues on SURF4 are important for the delivery of BMP8A and SFRP1 from the ER to the Golgi. Expression of an siRNA-resistant construct of SURF4 (SURF4^WT,RS^-HA) rescued the kinetic delay in delivery of SBP-EGFP-ShhN, SBP-EGFP-BMP8A, and SBP-EGFP-SFRP1 to the Golgi in SURF4 knockdown cells (Fig. 2). Around 60% of SURF4 knockdown cells expressing the rescue construct showed juxta-nuclear–localized SBP-EGFP-ShhN, SBP-EGFP-BMP8A, and SBP-EGFP-SRRP1 (Fig. 5, *A*–*C*, *H–J* and *O–Q* and quantifications in Fig 5, *G*, *N* and *U*). By contrast, the percentage of cells showing juxta-nuclear–localized cargo proteins was significantly lower in cells expressing an siRNA-resistant construct of SURF4 bearing E50A, D53A, and D56A mutations (SURF4^MT,RS^-HA) (Fig. 5, *D*–*F*, *K–M* and *R–T* and quantifications in Fig. 5, *G*, *N* and *U*). This analysis suggests that the E50, D53, and D56 residues on SURF4 are important for the delivery of polybasic motif–containing cargo proteins from the ER to the Golgi.

### The interaction between SURF4 and the polybasic motif is sufficient to promote ER-to-Golgi trafficking

**Figure 6:**
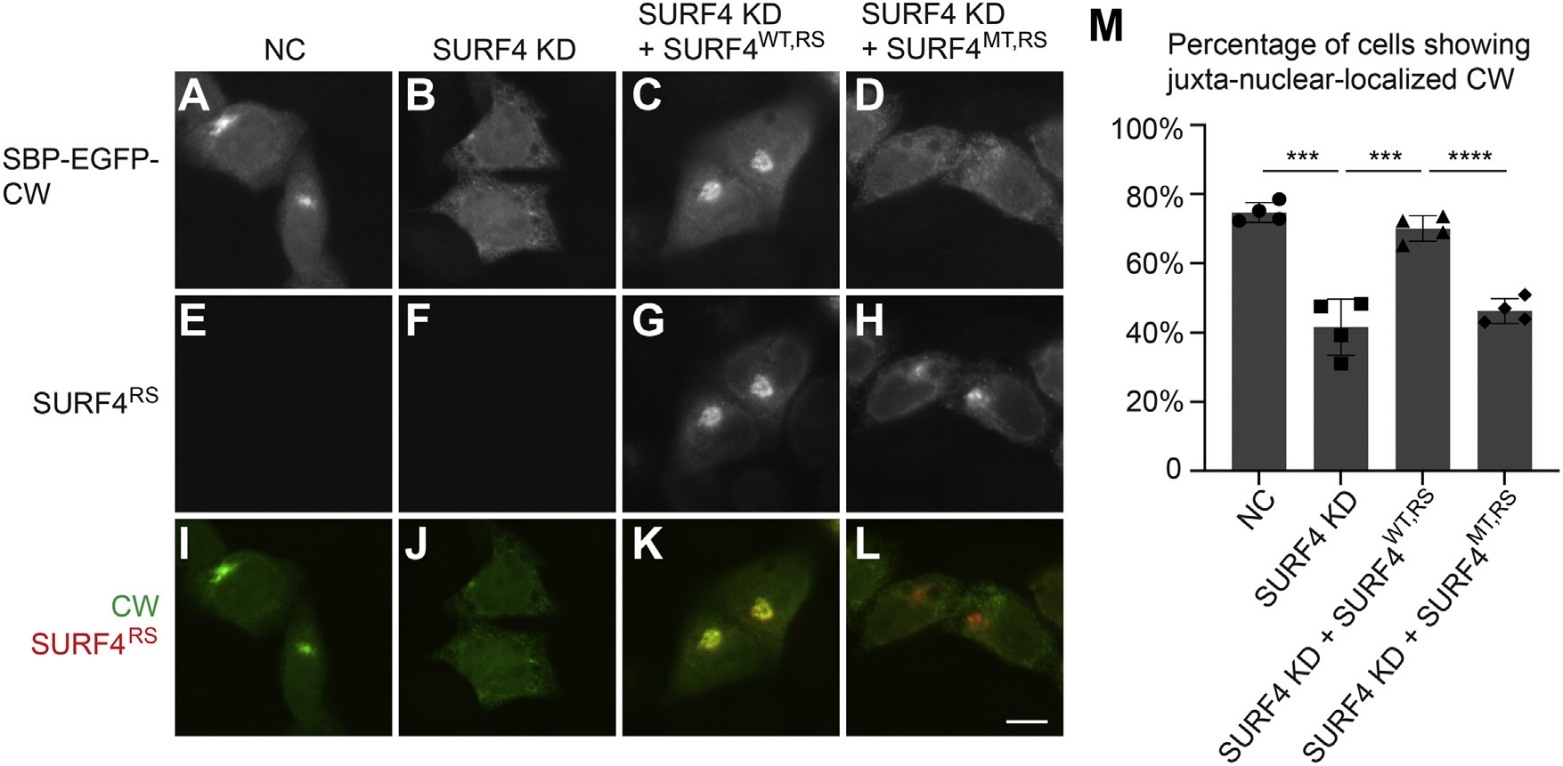
**The interaction between SURF4 and the polybasic motif is sufficient to promote ER-to-Golgi trafficking.***A*–*L*, HeLa cells were transfected with negative control (NC) siRNA (*A*, *E* and *I*) or siRNA against SURF4 (*B*–*D*, *F*–*H* and *J*–*L*). At 48 h after transfection, cells were retransfected with plasmids encoding Str-KDEL_SBP-EGFP-CW (*A*–*D*) or cotransfected with siRNA-resistant SURF4^WT,RS^-HA and Str-KDEL_SBP-EGFP-CW (*E*–*H*) or cotransfected with siRNA-resistant SURF4^MT,RS^-HA and Str-KDEL_SBP-EGFP-CW (*I*–*L*). On day 3 after knockdown, cells were incubated with biotin for 15 min, and the localization of the RUSH proteins was analyzed (The scale bar represents 10 μm). Magnification, 63×. (*M*), quantification of the percentage of cells showing juxta-nuclear–accumulated EGFP signal (mean ± SD; n = 4; >50 cells counted for each group). ∗∗∗*p* < 0.001; ∗∗∗∗*p* < 0.0001. CW, Cardin–Weintraub; EGFP, enhanced GFP; ER, endoplasmic reticulum; RUSH, Retention Using Selective Hook; SBP, streptavidin-binding peptide; SURF4, surfeit locus protein 4.

Our results indicate that the interaction between the triacidic motif of SURF4 and the polybasic motif is necessary to regulate the ER-Golgi trafficking of several polybasic motif–containing secretory proteins. We then analyzed whether the SURF4–polybasic motif interaction is sufficient for SURF4-mediated ER export. A RUSH construct without the cargo protein sequence (SBP-EGFP) was retained in the ER in the majority of cells after biotin treatment (16). In contrast, a RUSH construct composed of the polybasic CW motif of Shh fused to SBP-EGFP (SBP-EGFP-CW) was delivered from the ER to the Golgi after biotin treatment in ∼75% of cells (Fig. 6*A* and quantification in Fig. 6*M*). Thus, the CW motif is sufficient for exporting SBP-EGFP out of the ER efficiently. Consistent with our previous report (16), knockdown of SURF4 caused a kinetic delay in delivery of SBP-EGFP-CW to the Golgi (Fig. 6*B* and quantification in Fig. 6*M*). Here, we found that this delay was rescued by SURF4^WT,RS^-HA but not by the expression of SURF4^MT,RS^-HA (Fig. 6, *C* and *D*, and quantification in Fig. 6*M*). These analyses suggest that the interaction between the triacidic motif on SURF4 and the polybasic motif on cargo proteins is sufficient to promote ER-to-Golgi trafficking.

### SURF4 localizes at a subpopulation of ERESs to mediate ER export

**Figure 7:**
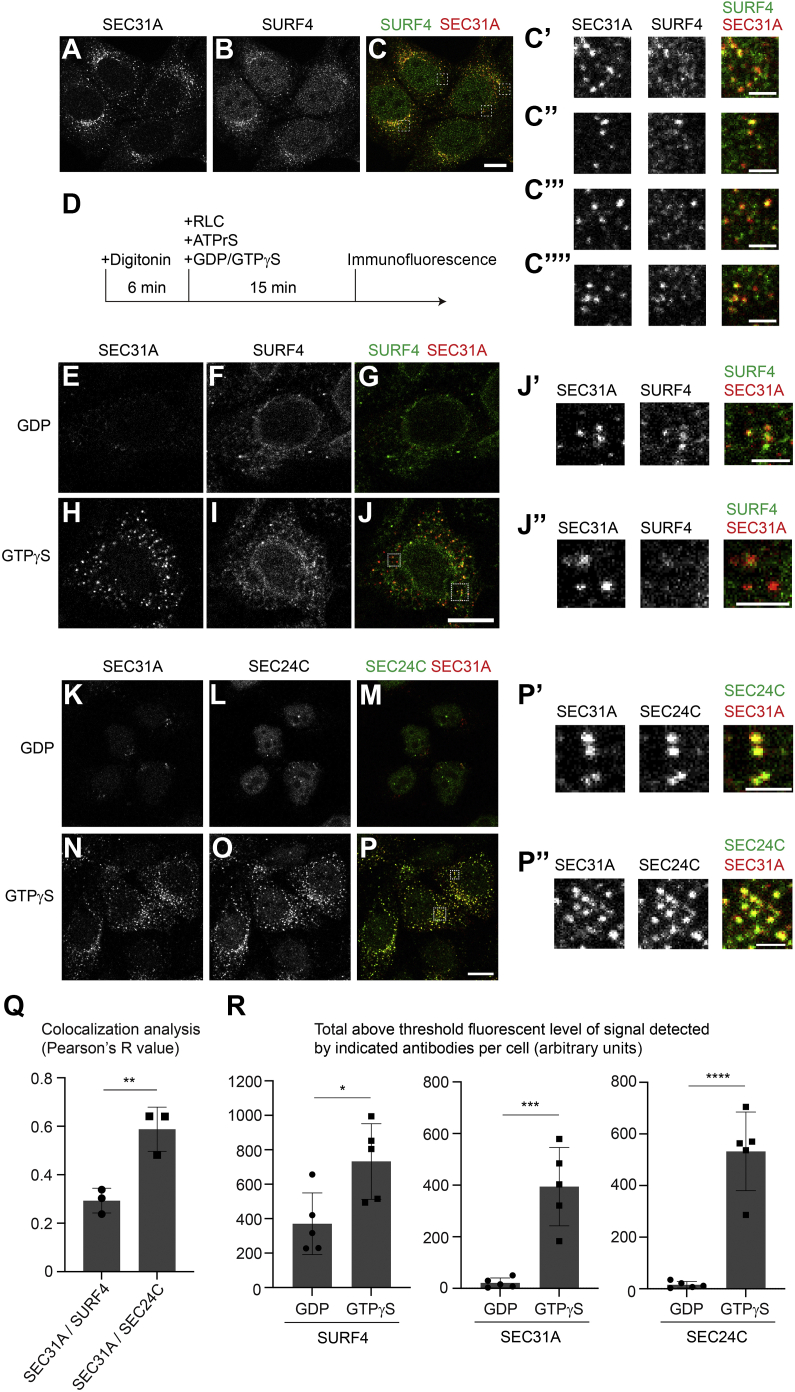
**Analysis of the colocalization between SEC31A and SURF4 or SEC24C.***A*–*C*, the localizations of SEC31A and SURF4 were analyzed in HeLa cells (The scale bar represents 10 μm). Magnified views of the indicated areas in panel C are shown in panels *C’–C’’’’* (The scale bar represents 2 μm). *D*, diagram depicting permeabilized cell assay. *E*–*P*, HeLa cells were permeabilized by digitonin and incubated with the indicated reagents. After incubation, the localizations of the indicated proteins were analyzed by immunofluorescence (The scale bar represents 10 μm). Magnification, 63×. Magnified views of the indicated areas in panels *J* and *P* are shown in panels *J’*, *J’’*, *P’* and *P’’* (The scale bar represents 2 μm). *Q*, quantification of the colocalization between SEC31A and SURF4 or SEC24C (n = 3, mean ± S.D., over 25 cells from five random imaging fields were quantified in each experimental group). ∗∗*p* < 0.01. *R*, quantifications of the total above-threshold fluorescent level of the signal detected by antibodies against SURF4, SEC31A, or SEC24C in each cell (mean ± SD; five random fields per group are quantified; >20 cells were quantified in each field). One representative experiment from three biological repeats is shown. ∗*p* < 0.05; ∗∗∗*p* < 0.001; ∗∗∗∗*p* < 0.0001. SURF4, surfeit locus protein 4.

Next, we performed an siRNA knockdown experiment to reduce the expression of SAR1A and SAR1B (24). Knockdown of SAR1A and SAR1B significantly reduced the efficiency of ER-to-Golgi transport of SBP-EGFP-BMP8A and SBP-EGFP-SFRP1 (Fig. S2), indicating that the ER exit of BMP8A and SFRP1 is COPII-dependent. To further investigate how the cargo receptor SURF4 regulates ER export of polybasic motif–containing cargo proteins, we analyzed the localization of endogenous SURF4 and COPII coat subunit SEC31A. Interestingly, we found that the punctate structures of SURF4 were colocalized with a subpopulation of ERES marked by the outer COPII subunit SEC31A at the steady state in HeLa cells (Fig. 7, *A*–*C*, magnified views in Fig. 7, *C’–C’’’’*). We then performed a permeabilized cell assay to lock the ER export process at the cargo sorting stage (16) and analyzed the localization between SURF4 and the inner COPII coat subunit SEC24C and the outer COPII coat subunit SEC31A. HeLa cells were permeabilized by mild detergent digitonin. The semi-intact cells were then washed with the high-salt buffer to remove the endogenous cytosolic proteins and then incubated with rat liver cytosol (RLC) and ATP regeneration system (ATPrS) in the presence of GDP or GTPγS. At 15 min after incubation, the localization of endogenous SURF4, SEC24C, and SEC31A was analyzed (Fig. 7*D*). When the semi-intact cells were incubated in the presence of GDP, SURF4 was partially located in peripheral punctate structures and partially located at the ER (Fig. 7*F*). SEC31A and SEC24C showed a weak signal (Fig. 7, *E* and *K*–*M*). When permeabilized cells were incubated in the presence of GTPγS, the total above-threshold fluorescent levels of SEC31A and SEC24C were significantly enhanced (Fig. 7, *H* and *N–P*, and quantification in Fig. 7*R*), indicating GTP-dependent recruitment of COPII at the ERES. We also detected a significant enhancement of the total above-threshold fluorescent level of SURF4 in the presence of GTPγS (Fig. 7*I* and quantification in Fig. 7*R*), suggesting that COPII assembly enhanced the recruitment of SURF4 at the ERES. SEC31A and SEC24C were recruited to punctate structures in the cell periphery and strongly colocalized with each other (Fig. 7, *N*–*P*, magnified views in Fig. 7, *P’* and *P’’*, and quantification in Fig. 7*Q*). We noticed that the punctate structures of SURF4 were colocalized with a subpopulation of SEC31A punctate structures similar to the intact cells (Fig. 7, *H*–*J*, magnified views in Fig. 7, *J’* and *J’’*). Quantification analyses indicated that the colocalization between SURF4 and SEC31A was significantly lower than that between SEC24C and SEC31A (Fig. 7*Q*). These analyses indicate that SURF4 is located on a subpopulation of ERES.

**Figure 8:**
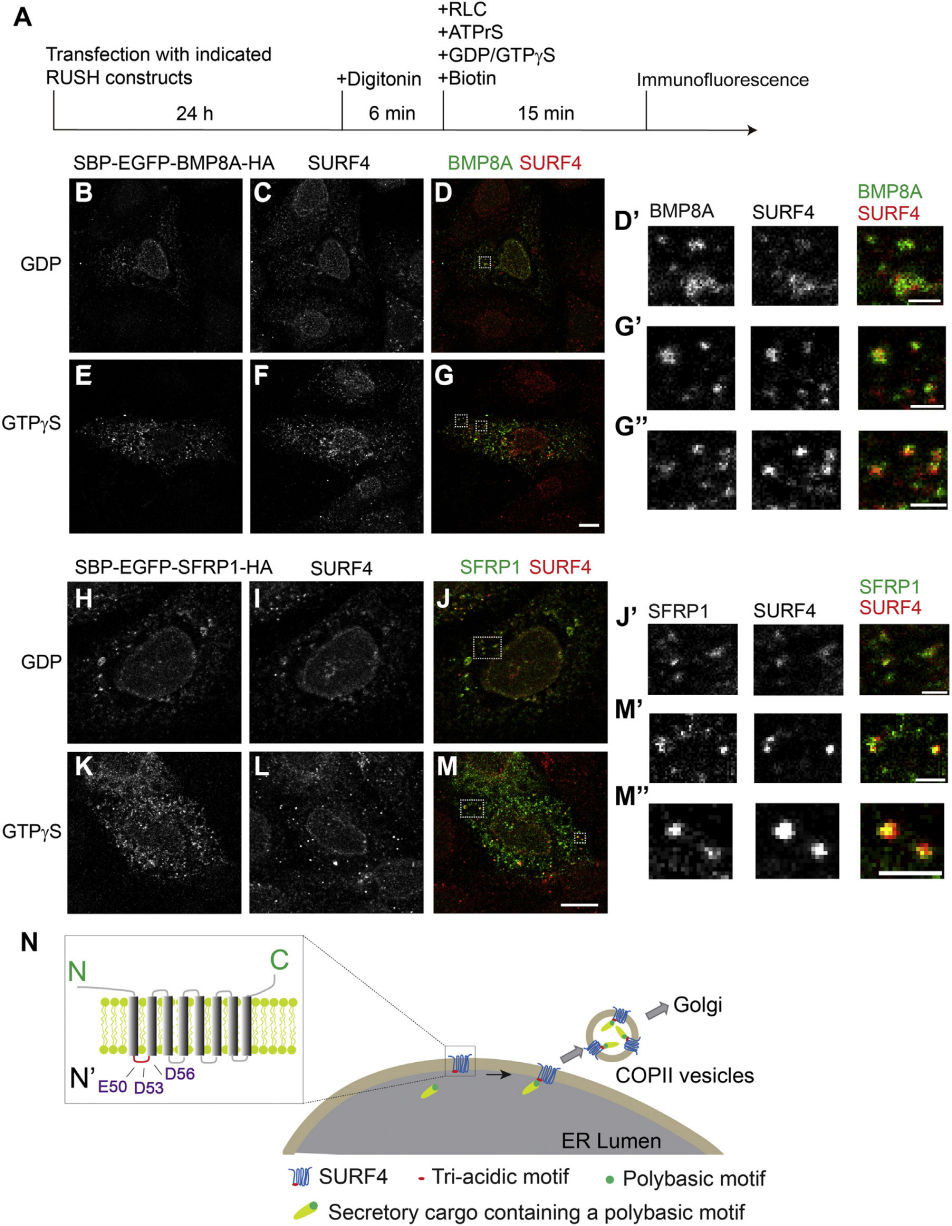
**Analysis of the colocalization between SURF4 and SBP-EGFP-BMP8A-HA or SBP-EGFP-SFRP1-HA using a permeabilized cell assay.***A*, diagram depicting permeabilized cell assay. *B*–*M*, HeLa cells were transfected with the indicated RUSH constructs. Day 1 after transfection, cells were permeabilized by digitonin and incubated with the indicated reagents. After incubation, the localization of the indicated proteins was analyzed by immunofluorescence (The scale bar represents 10 μm). Magnification, 63×. Magnified views of the indicated areas in panels *D*, *G*, *J* and *M* are shown in panels *D’*, *G’*, *G’’*, *J’*, *M’* and *M’’*, respectively (The scale bar represents 2 μm). *N*, our proposed model depicting the molecular mechanisms that SURF4 regulates the ER export of polybasic motif–containing secretory proteins. Magnified view of the indicated areas is shown in panels N’. BMP8A, bone morphogenetic protein 8A; EGFP, enhanced GFP; ER, endoplasmic reticulum; RUSH, Retention Using Selective Hook; SBP, streptavidin-binding peptide; SURF4, surfeit locus protein 4.

We next utilized the permeabilization assay to analyze the colocalization between SURF4 and its cargo proteins, BMP8A and SFRP1. HeLa cells expressing RUSH constructs of BMP8A or SFRP1 were permeabilized by digitonin and incubated with RLC, ATPrS, and biotin in the presence of GDP or GTPγS. After incubation, immunofluorescence was performed to analyze the localization of SBP-EGFP-BMP8A-HA, SBP-EGFP-SFRP1-HA, and SURF4 (Fig. 8*A*). We detected punctate localization of SBP-EGFP-BMP8A-HA and SBP-EGFP-SFRP1-HA in the cell periphery in the presence of either GDP or GTPγS (Fig. 8, *B*, *E*, *H* and *K*). Many of the punctate structures colocalized with SURF4 (Fig. 8, *B*–*M*, magnified views in Fig. 8, *D’*, *G’*, *G’’*, *J’*, *M’* and *M’’*). These analyses indicated that SURF4 was located on a subpopulation of ERES to mediate ER export.

## Discussion

In this study, we demonstrated that the electrostatic interactions between the triacidic motif of SURF4 and the polybasic motifs of BMP8A and SFRP1 are necessary for ER-to-Golgi trafficking of these SURF4 clients. We previously reported that SBP-EGFP represents an inert reporter with no endogenous export signals (16). Adding the polybasic CW motif of Shh to this inert reporter is sufficient to promote ER export (16). Here, we demonstrated that the triacidic motif of SURF4 is important for ER export of this construct, suggesting the electrostatic interaction between SURF4 and the polybasic motif is sufficient to drive SURF4-dependent ER export. We hypothesized that the triacidic motif of SURF4 allows SURF4 to recognize secretory cargo proteins bearing polybasic patches on their surface (Fig. 8*N*). Although this mode of cargo recognition has low specificity, it expands the repertoire of SURF4 clients. In addition to polybasic motif–containing cargoes, SURF4 also mediates ER export of other soluble proteins, including lipoproteins, proprotein convertase subtilisin/kexin type 9, and N-terminal hydrophobic-proline-hydrophobic motif–containing cargoes (8, 13, 15). It is currently unclear whether the N-terminal tripeptide motif interacts with SURF4 directly or indirectly. It is possible that the polybasic motif and the N-terminal tripeptide motif directly interact with different sites on SURF4. Another possibility is that SURF4 indirectly interacts with the N-terminal tripeptide motif through an unknown cellular factor.

Secretory cargo molecules are also implicated in entering COPII vesicles by bulk flow independent of receptors or sorting signals (1). Our previous report and current study indicated that the polybasic motif is necessary and sufficient to promote ER-to-Golgi trafficking of secretory proteins including Shh, Ihh, Dhh, BMP8A, and SFRP1, suggesting that bulk flow is not an efficient way to export these cargo proteins out of the ER. Apart from SURF4-mediated ER export, ER-to-Golgi trafficking of SURF4 clients can also be regulated by other factors. Unfolded SURF4 clients can associate with proteins in the ER quality control machinery to be retained in the ER. After ER export, SURF4 clients may also be recognized by COPI coat to be retrieved back to the ER. The outcome of ER retention, ER export, and ER retrieval will determine the efficiency of ER-to-Golgi trafficking of these soluble cargo proteins.

How is SURF4 disengaged with its clients at the destination? We previously showed that proteoglycans, which are synthesized at the Golgi, compete with SURF4 to interact with the CW motif of Shh, causing dissociation of Shh from SURF4 at the Golgi (16). The CW motif showed a consensus sequence of φBBφBφ or BBBφφBB, where B is a basic residue or rarely a histidine residue and φ is a hydrophobic residue (25). The polybasic motif of many of the identified secretory cargo proteins (Fig. 1*A*) do not contain hydrophobic residues between the basic residues. It remains unknown whether the polybasic motifs of BMP8A and SFRP1 interact with proteoglycans and whether this interaction is important to cause their dissociation from SURF4 at the Golgi. It is also possible that other protein factors or low pH can induce the dissociation of these SURF4 clients from SURF4 at the Golgi.

The results from our digitonin-permeabilized cell assay indicated that SURF4 showed punctate localization patterns in the presence of GDP (Figs. 7*F* and 8, *C* and *I*). We hypothesize the punctate localization pattern is caused by enrichment of SURF4 at the ERES, which takes place independent of the assembly of the COPII coat. Moreover, we found that some of the punctate structures of SURF4 colocalized with its clients in the presence of GDP, suggesting that SURF4 recognizes its clients independent of the COPII coat. This assay also indicated that the enrichment of SURF4 at the punctate structures is enhanced in the presence of GTPγS, indicating that the assembly of COPII coats enhances the enrichment of SURF4 at the ERES. Interestingly, we found that SURF4 was located in a subpopulation of ERES marked by the outer COPII coat subunit SEC31A. A possible explanation of this observation is that the assembly of COPII and the enrichment of SURF4 at the ERES are two separate events. Thus, some of the assembled COPII may not contain SURF4. Another possibility is that some of the ERES marked by COPII are occupied by other cargo receptors, which may compete with SURF4 to be associated with the COPII machinery.

We demonstrated that SURF4 has a capacity to mediate the secretion of polybasic motif–containing secretory cargo proteins. Many of these secretory proteins, such as Shh, induce cellular processes that promote cancer progression (26). Thus, downregulating the SURF4-mediated secretion of these secretory proteins provides a therapeutic strategy for cancer treatment. The electrostatic interactions important for SURF4-driven ER export provide crucial information to design inhibitors to downregulate SURF4-mediated protein secretion.

## Experimental procedures

### Constructs, reagents, cell culture, transfection, and immunofluorescence

HeLa cells and HEK293T cell lines were kindly provided by the University of California, Berkeley Cell Culture Facility and confirmed by short tandem repeat profiling. All cell lines were tested negative for *mycoplasma* contamination. HeLa and HEK293T cells were cultured in Dulbecco’s Modified Eagle Medium containing 10% fetal bovine serum and 1% penicillin–streptomycin mix (Invitrogen).

The cDNAs encoding WT and polybasic motif–mutated mouse ShhN, human DhhN, human IhhN, human BMP8A and human SFRP1, and WT human SURF4 were ordered from BGI Genomics. Standard molecular cloning procedures were performed to generate the plasmids encoding C-terminal 3 × HA-tagged ShhN (aa:1–198), C-terminal 3 × HA-tagged BMP8A, C-terminal 3 × HA-tagged SFRP1, C-terminal 3 × HA-tagged SURF4, N-terminal GST-tagged SURF4^49–60^, Str-KDEL_SBP-EGFP-ShhN (aa: 25–198), Str-KDEL_SBP-EGFP-DhhN (aa: 23–198), Str-KDEL_SBP-EGFP-IhhN (aa:28–202), Str-KDEL_SBP-EGFP-BMP8A (aa:20–402), Str-KDEL_SBP-EGFP-SFRP1 (aa:23–314), Str-KDEL_SBP-EGFP-CW, the C-terminal 3 × HA-tagged RUSH constructs (Str-KDEL_SBP-EGFP-BMP8A-HA, Str-KDEL_SBP-EGFP-SFRP1-HA), and the plasmids encoding truncated or mutated versions of ShhN, BMP8A, and SFRP1. The N-terminus of the SBP-EGFP tag was followed by a signal sequence derived from IL-2 (17). The plasmids encoding siRNA-resistant SURF4-HA were generated by QuickChange II site-directed mutagenesis using a plasmid encoding SURF4-HA as a template.

siRNA against SURF4 was purchased from RiboBio Co, Ltd. The target sequence is GCAGGAACTTCGTGCAGTA. The commercial antibodies were rabbit anti-HA (Cell Signaling Technology, catalog number 3724), mouse anti-HA (BioLegend, catalog number 901501), sheep anti-TGN46 (Bio-Rad Laboratories, catalog number AHP500G), mouse anti-SEC31A (BD Biosciences, catalog number 612350), and rabbit anti-SEC24C (Abcam, catalog number ab122633). Rabbit anti-SURF4 antibodies were kindly provided by Prof. Xiaowei Chen (Peking University). Rabbit anti-GFP antibodies were kindly provided by Prof. Robert Qi (Hong Kong University of Science and Technology).

Transfection of siRNA or DNA constructs into HeLa cells or HEK293T cells and immunofluorescence were performed as described previously (16). Images were acquired with Eclipse Ti Motorized Inverted Fluorescence Microscope (Nikon) equipped with an Andor Zyla 4.2 sCMOS camera (Andor Technology) or Leica SP8 Confocal Laser Scanning Microscope (Leica).

### RUSH assay and permeabilized cell assay

RUSH assays were performed by treating HeLa cells transfected with plasmids encoding Str-KDEL and different versions of SBP-EGFP-ShhN, SBP-EGFP-DhhN, SBP-EGFP-IhhN, SBP-EGFP-BMP8A, or SBP-EGFP-SFRP1 in a complete medium containing 40 μM biotin (Sigma-Aldrich) and 100 ng/μl cycloheximide (Sigma-Aldrich) for the indicated time. Cells were then fixed by 4% paraformaldehyde and mounted on glass slides by ProLong Gold Antifade Mountant with DAPI (Invitrogen) for microscope analysis.

Permeabilized cell assay was performed as described previously (27, 28). In brief, HeLa cells transfected with Str-KDEL_SBP-EGFP-BMP8A-HA or Str-KDEL_SBP-EGFP-SFRP1-HA were permeabilized by 0.04 mg/ml digitonin in KOAc buffer (110 mM KOAc, 2 mM Mg(OAc)_2_, 20 mM Hepes, pH 7.2) for 6 min at room temperature, followed by three washes in cold KOAc buffer. After 5 min of incubation on ice with cold 0.5 M KOAc buffer (0.5 M KOAc, 2 mM Mg(OAc)_2_, 20 mM Hepes, pH 7.2) followed by three washes in cold KOAc buffer to remove cytosolic proteins, the permeabilized cells were then incubated at 37 °C for 15 min in KOAc buffer containing 2 mg/ml RLC, 0.04 mM biotin, 500 μM GDP/GTPγS, and an ATPrS (40 mM creatine phosphate, 0.2 mg/ml creatine phosphokinase, and 1 mM ATP). The cells were then washed with cold KOAc buffer, fixed, and stained with specific antibodies.

### Protein purification and GST pull-down assay

Purification of GST-tagged SURF4^49–60^ was performed as described previously (29). GST pull-down assays were carried out with 10 μl of compact GSH beads bearing around 5 μg of GST-SURF4^49–60^. For binding experiments, the beads were incubated with 200 μl of 0.5 mg/ml cell lysates from HEK293T cells transfected with WT or truncated versions of ShhN-HA, BMP8A-HA, or SFRP1-HA in KOAc buffer with mixing at 4 °C overnight. After incubation, the beads were washed three times with 500 μl of 0.5 M KOAc buffer and three times with 500 μl of KOAc buffer, and the bound material was analyzed by immunoblotting.

## Data availability

All data are contained within the article and accompanying supporting information.

## Supporting information

This article contains supporting information.

## Conflict of interest

The authors declare that they have no conflicts of interest with the contents of this article.
