# Supplementary material for: Export of polybasic motif–containing secretory proteins BMP8A and SFRP1 from the endoplasmic reticulum is regulated by surfeit locus protein 4
Source: J Biol Chem. 2022 Nov 9;298(12):102687. doi: 10.1016/j.jbc.2022.102687 (PMC9731852; doi:10.1016/j.jbc.2022.102687)
Supplement: Supplemental Figures S1 and S2 [file mmc1.pdf]

Figure S1

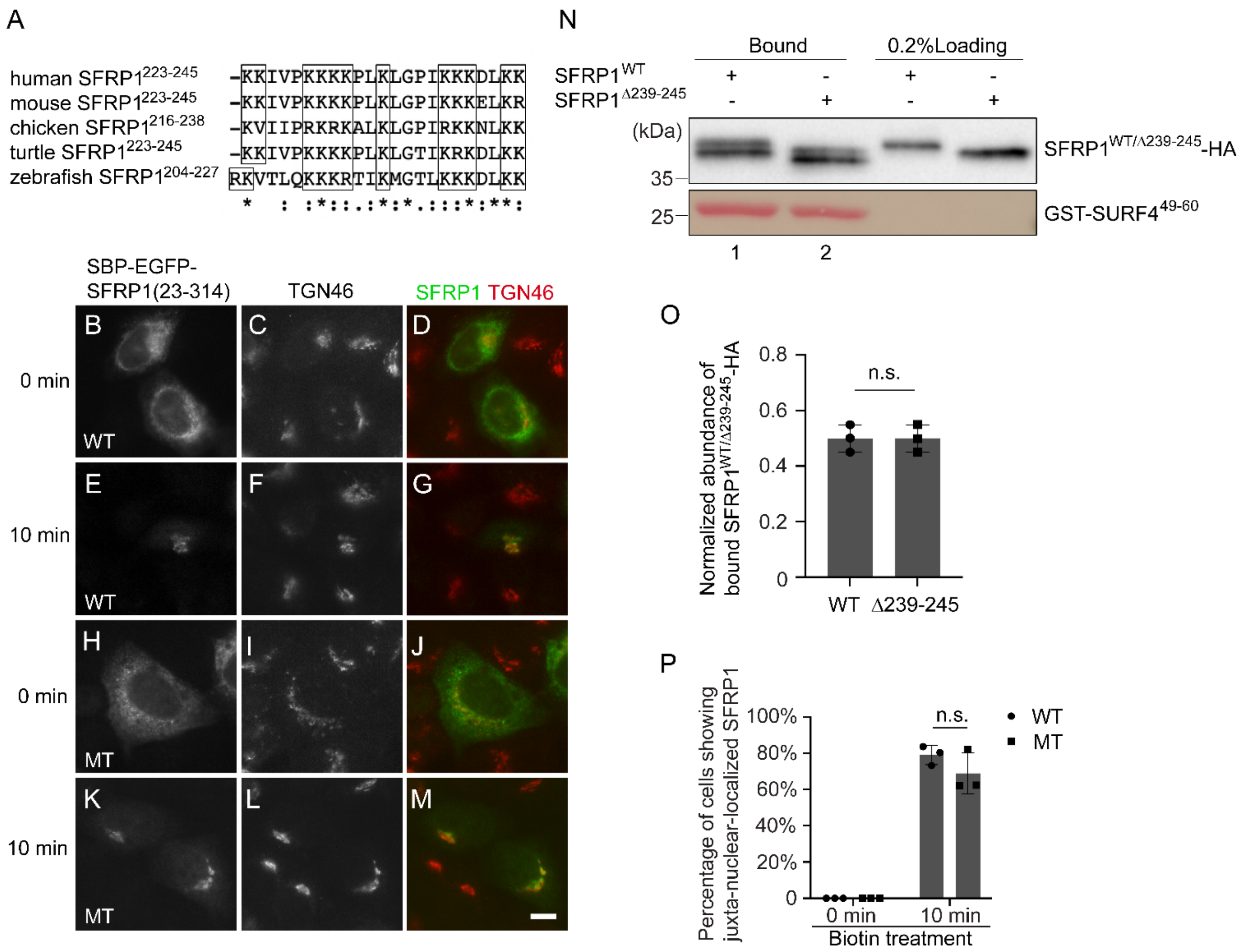

Fig. S1. Depleting (K/R)(K/R)(K/R)XX(K/R)(K/R) motif on SFRP1 did not cause defects on ER-to-Golgi transport of SFRP1. (A) Sequence alignment of SFRP1 across species. (B-M) HeLa cells were transfected with plasmids encoding Str-KDEL and SBP-EGFP-SFRP1 (B-G) or (K/R)(K/R)(K/R)XX(K/R)(K/R) motif mutated version of SFRP1 (H-M). Day 1 after transfection, the localizations of the different versions of RUSH constructs were analyzed after incubation with biotin for the indicated time (scale bar, 10 µm). Magnification, 63×. (N) Purified GST-tagged human SURF4$^{49-60}$ was incubated with lysates from HEK293T cells transfected with plasmids encoding wild-type or (K/R)(K/R)(K/R)XX(K/R)(K/R) motif-depleted SFRP1-HA. After incubation, the bound proteins were analyzed by immunoblotting with anti-HA antibodies. (O) Normalized abundances of SFRP1-HA bound to GST-SURF4$^{49-60}$ were quantified (mean ± SD; n = 3). The abundance of bound proteins was normalized to the bait protein GST-SURF4$^{49-60}$, and this value was then normalized to the sum of the normalized abundance of wild-type SFRP1-HA and mutant SFRP1-HA bound to GST-SURF4$^{49-60}$ in each experimental group. n.s., not significant. (P) Quantification of the percentage of cells showing juxta-nuclear-accumulated EGFP signal after incubation with biotin for the indicated time (mean ± SD; n = 3; >50 cells counted for each time point). n.s., not significant.

Figure S2

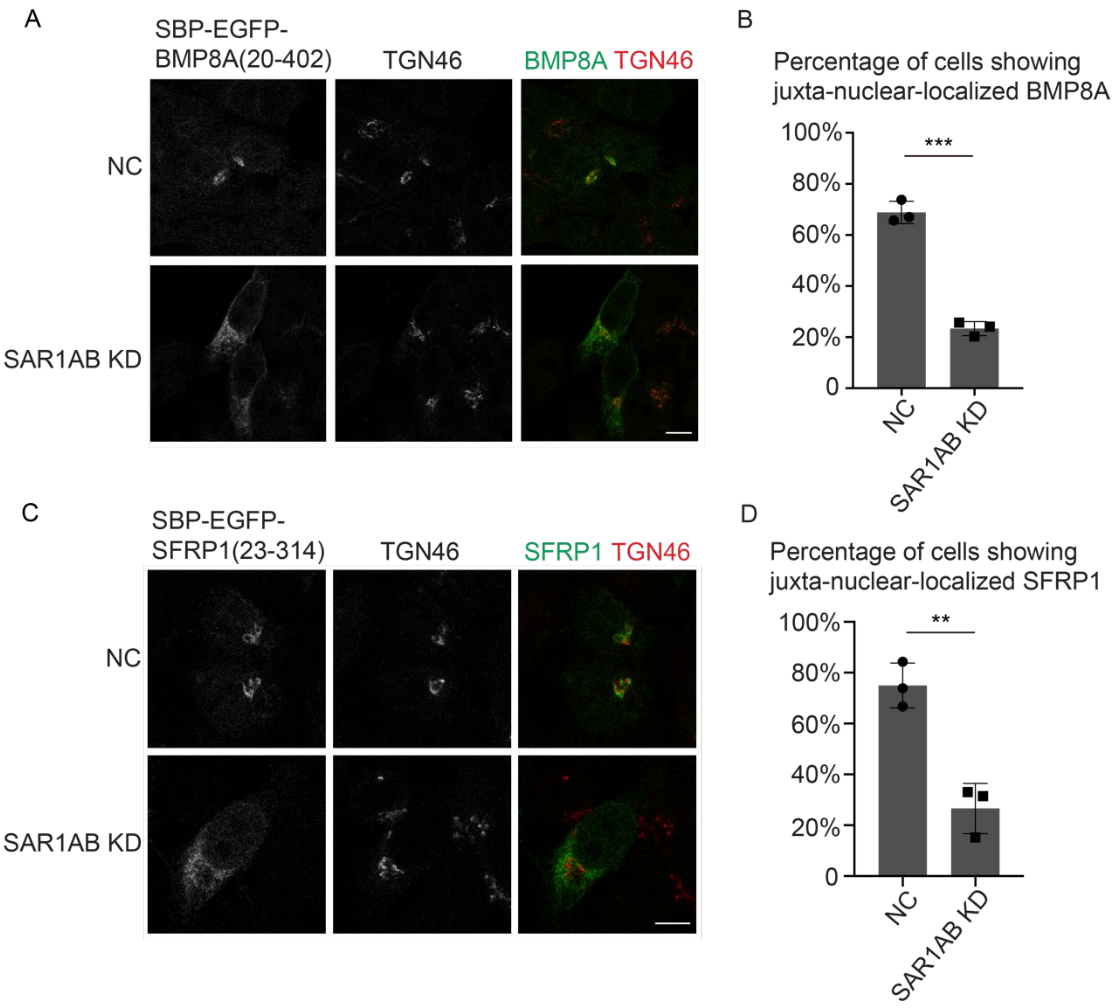

Figure S2. The ER-to-Golgi transport of BMP8A and SFRP1 is COPII-dependent. (A) and (C) HeLa cells were transfected with negative control (NC) siRNA or siRNA against SAR1A and SAR1B. At 48 h after transfection, cells were re-transfected with plasmids encoding Str-KDEL_SBP-EGFP-BMP8A (A) and Str-KDEL_SBP-EGFP-SFRP1 (C). On day 3 after knockdown, cells were incubated with biotin for 30 min (A) or 8 min (C), and the localization of BMP8A and SFRP1 was analyzed (scale bar, 10 μm). Magnification, 63×. (B) and (D) Quantifications of the percentage of cells showing juxta-nuclear-accumulated EGFP signal (mean ± SD; n = 3; >100 cells counted for each group). $^{**}P < 0.01$; $^{***}P < 0.001$.
